# Exercise reduces systemic immune inflammation index (SII) in childhood cancer patients

**DOI:** 10.1007/s00520-021-06719-3

**Published:** 2021-12-03

**Authors:** Matteo Winker, Sandra Stössel, Marie Astrid Neu, Nadine Lehmann, Khalifa El Malki, Claudia Paret, Niklas Joisten, Wilhelm Bloch, Philipp Zimmer, Jörg Faber

**Affiliations:** 1 Department for Molecular and Cellular Sports Medicine, Institute of Cardiovascular Research and Sports Medicine, German Sport University, Cologne, Germany; 2 Center for Pediatric and Adolescent Medicine, Childhood Cancer Center, University Medical Center Mainz, Mainz, Germany; 3 Department of “Performance and Health (Sports Medicine)”, Institute of Sport and Sport Science, Technical University Dortmund, Otto-Hahn-Straße 3, 44227 Dortmund, Germany

**Keywords:** Tumor entities, Cellular inflammation, Pediatric oncology, Resistance training, Endurance training

## Abstract

While exercise and physical activity have been suggested to reduce mortality and symptoms in cancer, knowledge on these associations in patients with childhood cancer (CCPs) is sparse. Anti-inflammatory properties of exercise might mediate these beneficial effects. We investigated the influence of exercise on the inflammation markers neutrophil-to-lymphocyte ratio, platelet-to-lymphocyte ratio, and systemic-immune-inflammation index (SII) and associations to patient-reported-outcomes in CCPs in a randomized-controlled trial. Results show associations between inflammation markers and patient-reported outcomes. Compared to the control group, SII was significantly reduced following exercise (*p*=0.036). Anti-inflammatory effects of exercise are also present in CCPs and may underlie exercise-induced benefits on symptoms. Clinical Trial Registration Number: NCT02612025

## Introduction

In adults, exercise and physical activity reduce cancer mortality [1] as well as treatment-related side effects, such as depression or fatigue [2, 3]. Some of these beneficial properties have been suggested to be mediated by the anti-inflammatory environment which is promoted through regular exercise [1, 4]. Recently, research approaches have tried to transfer these promising findings to the childhood cancer setting [5, 6].

Inflammation contributes to different chronic diseases and is associated with several disease-specific symptoms in patients with childhood cancer (CCPs) [7, 8]. In the past years, the cellular immune inflammation markers neutrophil-to-lymphocyte ratio (NLR), platelet-to-lymphocyte ratio (PLR), and systemic immune-inflammation index (SII) have been proofed to be reliable determinants of inflammatory responses. Indeed, several studies confirmed the prognostic value of all three markers with regard to progression [9], symptoms [3], and survival [1] in different cancer diseases. These promising and easily traceable markers were shown to be reduced by exercise in patients with multiple sclerosis [10].

Since knowledge on the benefits of exercise training in CCPs is sparse, an investigation of potentially underlying mechanisms would be helpful to improve population-specific exercise recommendations. Here, we exploratory investigated potential associations of NLR, PLR, and SII, with disease- and treatment-related symptoms and whether an exercise intervention decreases these markers in CCPs. We hypothesize that NLR, PLR, and SII are associated with different symptoms such as fatigue and quality of life (QoL) and that an exercise intervention decreases these markers in CCPs.

## Methods

In this secondary analysis of a randomized controlled trial, NLR, PLR, and SII from 25 CCPs undergoing cancer treatment aged 4–17 years (Table 1) were determined from venous blood samples. Participants were allocated to an exercise group (EG) or a control group (CG) using stratified randomization with age, sex, tumor entity, and physical performance status as stratification factors. The study was approved by the regional ethical committee. A detailed description of the primary study has been published elsewhere [11]. In brief, the EG participated in a 6–8-week supervised exercise intervention including three exercise sessions per week (45–60min) of patient-adapted, moderate intensity endurance and strength exercises. The timeframe of intervention varied since all exercise tests were conducted prior to a new cancer therapy cycle on condition of hematologic recovery to ensure comparability. Training load was adjusted based on the results of the 6-min walking test as well as a questionnaire asking for physical activity level and intensity in daily life, prior to disease onset [12]. During the intervention, intensity was considered as moderate if two of the following criteria were fulfilled: A score of 12 to 13 on the Borg Scale, 60–75% of estimated maximum heart rate, a maximum of three repetitions with correct technical execution, and a rating by the supervisor based on physiological parameters [13]. The CG received anti-cancer treatment recommended by their medical supervisor. To evaluate the level of fatigue, the PedsQL3.0 questionnaire was used [14]. The QoL was determined with the KINDL questionnaire [15]. Parent-report versions of both German language questionnaires were used. Venous blood samples were collected during a resting condition before the first exercise session (baseline) and after the last exercise session (post).

**Table 1:** Participants’ characteristics separated by exercise intervention

	EG (*n*=11)	CG (*n*=14)	Overall (*n*=25)	*p*-value
Sex (f/m)	5/6	7/7	12/13	-
Age at diagnosis (years)	11.19 ± 5.60	11.28 ± 4.41	11.24 ± 4.86	0.964
Bodyweight (kg)	46.00 ± 21.52	39.86 ± 17.97	42.56 ± 19.43	0.445
Tumor entity (AL or TCL/CNS/others)	6/1/4	5/2/7	11/3/11	-
6-min walk test (m)	450.17 ± 164.86	543.66 ± 76.16	502.52 ± 129.26	0.072
Fatigue at baseline (score)	61.53 ± 20.37	71.52 ± 7.43	67.03 ± 15.16	0.193
QoL at baseline (total score)	71.18 ± 8.76	71.91 ± 14.57	71.63 ± 12.39	0.893
Duration of treatment (weeks)	8.04 ± 1.45	8.42 ± 2.90	8.25 ± 2.32	0.700
Exercise sessions (numbers)	19.93 ± 8.85	-	-	-
NLR at baseline	1.54 ± 0.92	1.26 ± 1.09	1.39 ± 1.00	0.504
PLR at baseline	0.30 ± 0.20	0.33 ± 0.33	0.31 ± 0.27	0.825
SII at baseline	564.52 ± 412.43	737.22 ± 975.53	665.26 ± 782.31	0.605

*EG*, exercise group; *CG*, control group; *f*, female; *m*, male; *AL*, acute leukemia; *TCL*, T-cell lymphoma; *CNS*, central nervous system tumor; Fatigue [total score] evaluated with PedsQL 3.0 questionnaire by parents; QoL [total score] evaluated with KINDL questionnaire by parents; *NLR*, neutrophil-to-lymphocyte ratio; *PLR*, platelet-to-lymphocyte ratio; *SII*, systemic immune-inflammation index

Subjects were included if at least one blood sample was collected. NLR, SII, and PLR were calculated from hemograms using an automated hematology analyzer (Sysmex xs-800i von Sysmex Deutschland GmbH). Data was winsorized to *z*-scores of three [16] and tested for normality using Kolmogorov-Smirnoff test. Since normality could not be assumed for most of the variables, we log10 transformed NLR, SII, and PLR at both measurement time points. Spearman’s rank correlation coefficient was calculated to investigate potential associations between the cellular inflammation markers and quality of life and fatigue in CCPs. To evaluate the effects of the exercise intervention on NLR, SII, and PLR, delta values of EG versus CG were compared using a one-way analysis of variance (ANOVA). The level of significance was set at *p* ≤ 0.05. Statistical analysis was performed using IBM SPSS Statistics software Version 27 (IBM, Armonk, NY, USA).

## Results

**Fig. 1:**
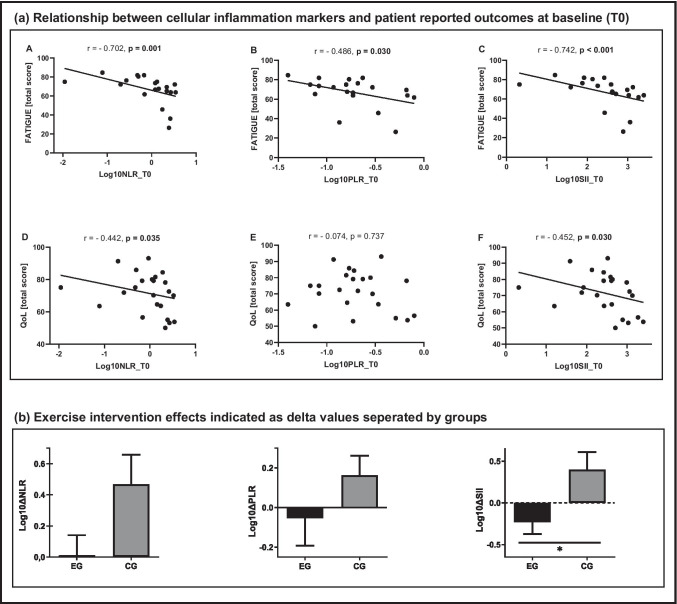
Correlation analysis (a) of fatigue with Log10-transformed data of (A) NLR, (B) PLR, (C) SII at baseline, as well as QoL with Log10-transformed data of (D) NLR, (E) PLR, and (F) SII at baseline. Log10-transformed data (b) of training effects from pre- (baseline) to post-intervention illustrated as delta values. NLR, neutrophil-to-lymphocyte ratio; PLR, platelet-to-lymphocyte ratio; SII, systemic immune inflammation index; Log10_T0, Log10-transformed data at baseline. Fatigue [total score] evaluated with PedsQL 3.0 questionnaire by parents; QoL [total score] evaluated with KINDL questionnaire by parents. Values are presented as mean ± standard error. *Significant between-group effect

Fatigue was negatively correlated with NLR (*r*=−0.702; *p*=0.001), PLR (*r* =−0.486; *p*=0.030), and SII (*r*=− 0.742; *p*<0.001) at baseline. QoL negatively correlated with NLR (*r*=−0.442; *p*=0.035) and SII (*r*=−0.452; *p*=0.030) at baseline (Fig. 1A). No significant correlation was observed between QoL and PLR (*r*=−0.074; *p*=0.737) at baseline. Graphical illustrations of associations between NLR, PLR, SII, and fatigue as well as QoL are depicted in Fig. 1A. Regarding exercise intervention effects, delta value comparisons showed a significant between group effect for SII (df=1, *F*=4.963, *p*=0.036). While the exercise intervention reduced SII, an increase was observed for the CG. No significant effects between the groups were observed for NLR (df=1, *F*=3.605, *p*=0.070) and PLR (df=1, *F*=0.484, *p*=0.493).

## Discussion

The results of this secondary analysis of the MUCKI trial confirm findings from studies in adults reporting associations between the cellular inflammation markers NLR, PLR, SII, and fatigue as well as QoL [3, 11, 17, 18]. Moreover, our results provide first evidence that a physical exercise intervention in CCPs was able to reduce SII.

From a descriptive point of view, the NLR shows an inverse development during the intervention period between the two groups, with a downregulation in the EG and an increase in the CG. However, statistical significance was only observed between the two groups for SII. The exercise-induced reduction of SII highlights the anti-inflammatory effects of exercise, which have been proposed to mediate the beneficial effects of exercise training on various symptoms in cancer patients [2, 19]. The present study suggests similar effects of exercise in CCPs.

The small sample size (*n*=25) and heterogeneity in included tumor entities represent central limitations of the investigation and may led to absent statistical between group differences of the NLR.

In conclusion, results of the present study confirm that the well-described anti-inflammatory effects of exercise are also present in CCPs [2, 5, 10]. Considering the exploratory character of this analysis, the reported findings cannot be generalized, and future powered randomized controlled trials are needed to confirm our promising findings and highlight the clinical relevance.
